# Reaching endpoints for lymphatic filariasis elimination- results from mass drug administration and nocturnal blood surveys, South Gujarat, India

**DOI:** 10.1371/journal.pntd.0005476

**Published:** 2017-04-03

**Authors:** Anjali Modi, Sukesha Gamit, Bharat S. Jesalpura, George Kurien, Jayendra K. Kosambiya

**Affiliations:** 1 Department of Community Medicine, Government Medical College, Surat, Gujarat, India; 2 National Vector Borne Disease Control Department (NVBDCP), Health and Family Welfare Department, Government of Gujarat, Gandhinagar, Gujarat, India; Walter and Eliza Hall Institute, AUSTRALIA

## Abstract

**Background:**

Following the World Health Assembly resolution on Elimination of lymphatic filariasis (ELF) as a public health problem by the year 2020, a Global Program (GPELF) was launched in 1997 to help endemic countries to initiate national programs. The current strategy to interrupt transmission of LF, is administration of once-yearly, single-dose, two-drug regimen (Albendazole with Diethylcarbamazine (DEC) to be used in endemic areas with the goal of reaching 65% epidemiological coverage for 4–6 years. We report findings of independent assessment from year 2010 to 2015 for last six rounds, after initial five rounds of Mass Drug Administration (MDA) since 2005 for ELF in endemic area of Gujarat.

**Methods:**

Independent assessment of MDA was performed to find coverage and compliance indicators, reasons for non-coverage and non-compliance in five Implementation Units (IUs). Pre, during and post MDA evaluations were done in three phases. The impact of MDA was measured by microfilaraemia survey. A total of eight sites, four random and four fixed sentinel sites were selected to calculate microfilaria rate (MF) per IUs per year. In years 2010 to 2015, we report results from 125,936 nocturnal blood smears and 17551 population in 120 selected clusters. Four clusters were selected per year in each of the five IUs for assessment of MDA round.

**Result:**

Post MDA survey showed drug coverage between 81%-88% and epidemiological coverage 77%-89% across years. Main reasons for non-coverage were drug administrator related (the team did not visit or missed people) while non-compliance was population related (fear of side effects, sickness, people forgot or absent). During MDA findings show that the directly observed consumption is considerably improved from 58% in 2010 to 82% in 2015. The knowledge about benefits of drug provided also increased from 59% to 90% over the years. The current MF rate is less than one in all IUs with an overall 68% percent decrease from baseline year 2005 to year 2015. The average MF rate of Gujarat is 0.44 for year 2015.

**Conclusions:**

The findings show that achieving adequate epidemiological and drug coverage is possible by actual field level operation of the program in large endemic areas. The results and feedback from independent assessment, performed regularly, could guide the policymakers and program managers for mid-term corrections and to frame strategies to enhance program. Monitoring of coverage and impact indicator together informs decisions for reaching end-point of MDA. The impact indicator- microfilaria rate in all IUs of South Gujarat Region has reached and remained less than one percent signaling end-points of MDA. Post MDA stringent monitoring in form of TAS is recommended to keep vigil on maintenance of elimination achieved.

## Author summary

The Global Program to Eliminate Lymphatic Filariasis (GPELF) is one of the largest public health intervention till date based on mass drug administration (MDA). MDA interrupts transmission by clearing larval parasites from blood so that they are unavailable for mosquitoes. The current communication describes independent assessment of annual MDA of diethylcarbamazine and albendazol in endemic area of Gujarat state, India during years 2010–2015. The process indicators coverage and compliance rates and impact indicator microfilaraemia were studied following WHO guidelines. We report results from 125,936 nocturnal blood smears and 17,551 population in 120 clusters. Post MDA survey shows high coverage and compliance above 85%. Both drug and epidemiological drug coverage were above recommended average of 80% and 65% respectively. Directly observed consumption improved from 58% in 2010 to 82% in 2015. The reports were shared with program managers and drug administrators to inform development of specific strategies to enhance the program. Microfilaraemia decreased by 68% from baseline year 2005 to 2015. Findings demonstrate implementing MDA gives sustainable results in large populations. After ten rounds MDA, LF transmission is interrupted and microfilaraemia decreased to <1%. Now, Gujarat State is ready for post-MDA surveillance and Transmission Assessment Survey (TAS).

## Introduction

Worldwide 947 million people are at risk of lymphatic filariasis (LF) infection in 54 countries [1, 2]. Following the World Health Assembly resolution, a Global Program on Elimination of LF (GPELF) by the year 2020, was launched in 1997 to help endemic countries initiate national programs [3].

India has about 40% of the global filariasis burden and 50% of the global population at risk of infection [4]. In India, 99.4% of filariasis infections are due to *Wuchereria Bancrofti* transmitted by the vector, *Culex quinquefasciatus* [5]. India started the annual mass drug administration (MDA) program to eliminate lymphatic filariasis (PLEF) in 11 endemic districts in 1997, on a pilot basis. By 2007 the program covered all 250 known endemic districts providing protection to entire 600 million people making it the largest national public health intervention [6, 7]. All LF endemic districts have done 9–11 rounds of MDA. The success of ELF is reflected in the decrease of overall microfilaria rate from 1.24% in 2004 to 0.44% in 2014 [8].

The current strategy to interrupt transmission of LF, is a once-yearly, single-dose, two-drug regimen (Albendazole with Diethylcarbamazine (DEC)) to be administered in communities [9] at risk with the goal of reaching 65% epidemiological drug coverage for 4–6 years [7, 10, 11]. The objective of MDA is to reduce the level of microfilaraemia in infected individuals so that transmission cannot be sustained, even after MDA has been stopped [11, 12].

Monitoring and Evaluation of MDA are vital to assess the impact of the program as well as to take evidence based decision to withdraw MDA [12]. The present WHO definitions do not account for the difference between receiving and consuming the drug [11]. As a result drug administrators (DAs) record the drugs they have distributed to the population rather than the number of drugs consumed. It has been observed in the past that actual drug consumption was lower than the reported coverage by DAs because a substantial proportion of community members do not consume the drug even if they receive it [11–13]. Therefore, it is important that the mid-term assessment is performed by an independent team not associated with the MDA program in the selected areas and the findings from the independent assessment can be used for programmatic improvement.

The available research on ELF and MDA are based on limited or selective populations therefore do not take into account the operational indicators and other outputs from a large scale Public Health Program implementation[4, 9, 14]. Also, none of the previous findings report process indicators coverage and compliance rate along with the impact indicator of Microfilaria rate to compare and understand the impact of MDA [4, 7, 9, 14–17].

The goal of this study is to perform an Independent Assessment of ELF program from 2010–2015 in endemic districts of Gujarat state in India, to ascertain the proportion of total and targeted population that received and ingested the drug every year. We aim to find epidemiological coverage, reasons for non-coverage and non-compliance and impact of MDA on microfilaria (MF%) rate in large geographical areas or whole Implementation units IUs. The findings from this study will inform policymakers and program managers for mid-course corrections and strengthening decisions for reaching end-point of MDA by conducting TAS (Transmission Assessment Surveys).

## Materials and methods

### Study settings

The MDA round has been on going in Gujarat state since 2004 [18]. The South Gujarat Region is a tropical region situated on the western coast of India between 21.17°N and 72.83°E. The area is 17500 sq km. and the population 9,919,499 according to the 2011 census. The region consists of districts Surat, Navsari, Tapi, Valsad and Surat city also known as Surat Municipal Corporation (SMC) which are endemic for filariasis.

### Study duration

The findings of independent assessment of MDA for ELF in endemic area of South Gujarat Region for the years 2010 to 2015 when five initial rounds were completed.

### Study design and methods

The MDA was planned and implemented by the Health Department of various districts and SMC. The MDA activity was supervised independently by faculties of Medical Colleges of Surat city which were not directly involved in the implementation of the health program. The independent assessment was done in three phases- Pre MDA, MDA and post MDA. Independent assessment was conducted in three phases: Pre MDA (assessment of preparedness), MDA (process or operational Research) and Post MDA (assessment of drug coverage and compliance).

The **Pre MDA** evaluation was performed in all IU for assessing the preparedness of the system in terms of implementation of the MDA.

**During MDA** Evaluation was done by direct observation of treatment (DOT) and exit interviews. Exit Interviews are interviews at the point of patients’ exit from a health care facility. They can be used for people’s utilization of public health services with a minimum recall period [19]. Twenty beneficiaries from each of the five IUs were selected to find about on-spot drug consumption, awareness and source of IEC for LF.

**Post MDA** Every year four survey clusters were selected per district (one urban and three rural) on basis of reported coverage for that year. The reported coverage of MDAs by DAs was used to classify Primary Health Centers (PHCs) in each district according to low, medium and high coverage. From each category, one PHC village was selected at random. One ward area of medium coverage was selected for urban. From each such cluster, 30 households which approximately had nearly 150 residents were surveyed. Accordingly, about 600 family members were interviewed in each of the five district/ IUs every year.

### Study participants and study tool

The people living in the endemic area/ at risk population for MDA were approached with a predesigned, pre-tested, structured questionnaire to record information about study variables. Head of the family or any other family member who had knowledge about family were interviewed. The eligible population did not include pregnant and lactating women, children below two years of age and seriously ill persons. Informal consent in form of oral consent was obtained from the participants.

### Bias

To assess actual compliance, in addition to recording oral responses for drug consumption, we also directly observed swallowing of drug (DOT) during all MDA rounds to reduce recall bias and respondent bias which was consistent for all years. All the members of survey team were trained every year to ensure uniformity in data collection. The study variables and findings from surveys of previous years were also discussed to reduce interviewer bias.

### Study variables

Primary outcomes were coverage and compliance of MDA. Various indicators recommended by WHO and research community were evaluated [11, 13, 14, 20]. Main indices were- coverage rate, compliance rate, coverage-compliance gap, drug coverage and epidemiological coverage rate. The definitions for indicator are provided in detail with denominator in Table 1.

**Table 1:** Definitions of study variables and outcomes.

Sr.No.	Indicator	Definition
1	At-risk population	Total population in the endemic implementation unit(s). It includes both eligible and ineligible population [20].
2	Target population for MDA	The population in an implementation unit that is targeted for treatment. This includes those who are eligible to receive the drugs based on safety criteria [11, 20].
3	Directly observed treatment (DOT) or (DOT-MDA)	The only method to ensure an individual swallowed a drug or a combination of drugs [20].
4	Coverage rate	Proportion of eligible population who received DEC & Albendazole [13].
5	Compliance rate	Proportion of people who consumed the drug out of those who received it [14].
6	Coverage Compliance gap	Proportion of those who received DEC but did not consume it [14].
7	Reported coverage	Intervention coverage calculated from data reported by all drug administrators [13, 20].
8	Drug (Effective) Coverage rate	The proportion of individuals, expressed as a percentage, in a targeted population who swallowed a drug, or a combination of drugs; the denominator is eligible/targeted population [11, 13,14, 20].
9	Epidemiological drug Coverage	The proportion of individuals in the implementation unit who have ingested the MDA drugs of the total population in the implementation unit; the denominator is total population [11, 13, 20].
10	Microfilaria rate (Mf%)	Number of slides positive for microfilaria from total number of slides examined [11, 12, 21].

The preparedness of ELF program and DOT-MDA was also assessed.

Coverage is the proportion of eligible people who received the antifilarial tablets [13]. Compliance is the proportion of people who actually ingested the tablets out of those who received it. The coverage—compliance gap is the proportion of people who receive the drugs but did not ingest them [14].

The drug coverage rate is proportion of eligible population who ingested the drug. The drug coverage in the targeted, or eligible, population is the best measure of how well MDAs were implemented. An adequate level of programme drug coverage is estimated to be 80% [11, 13, 20].

The ‘epidemiological drug coverage’ is the proportion of total population who ingested the drug. The epidemiological drug coverage among total population is a reflection of what proportion of the at-risk population is being covered by MDA. The operational definition of effective MDA is 65% coverage of total population.

The epidemiological drug coverage was the main outcome. The operational definition of effective MDA is 65% coverage of the total population [11]. The reasons for non-coverage and non-compliance were also assessed.

We also performed **a parasitological survey** to assess microfilaria rate (Mf %) which is the main impact indicator for MDA. A total of eight sites, four random and four fixed sentinel sites were selected per district. For each type of site- random or sentinel- three rural and one urban area were selected according to WHO guidelines [11]. The sentinel sites were sub-centers from three PHCs with the highest number of lymphedema and hydrocele cases detected during the morbidity survey for filariasis [12]. The sentinel sites remained the same every year since the Pre MDA Microfilaria survey. Every year four new areas were selected randomly as spot check sites. From each sentinel or random site, 500 night blood smears were collected between 8:30 PM and 12 midnight thus making a total of 4000 blood smears per district before each MDA round. The blood smear collection was done by the hanging drop method where 2–3 drops of blood were taken on a slide from third/ring finger of left hand. Another clean slide was taken to prepare a thick oval blood smear. Each slide was named and transported. The blood smears were dehaemoglobinised within 24 hours of collection, fixed and stained in JSB-1 stain [12]. The entire blood smear was examined systemically from one end to other for microfilariae. The species of microfilaria were identified using an oil immersion lens [21]. The microfilaria rate (Mf %) was calculated as the percentage of persons showing microfilaria in their peripheral blood (night blood smears) [11, 12, 21].

### Statistical analysis

The data was entered and analyzed in Microsoft Excel sheet. Simple descriptive statistics like frequency distributions were used to depict data.

### Ethical considerations

The data collection for this study was done as part of supervision of activities implemented for Elimination of Lymphatic Filariasis under National Vector borne Disease Control Program (NVBDCP). The supervision activity was independent but overall part of national Health program, therefore, ethical approval was not required. The participants provided oral informed consent to be part of interview. As per government regulation a written consent is not required for activities conducted under national public health programs considered beneficial for people. The oral consent was documented by names on data collection form but all identifiers were removed for data entry and collection. The forms were stored in a locked cabinet with access by principal investigator only.

## Results

The Pre MDA included training of staff, micro planning, Information Education and Communication (IEC) activity, supply and distribution of drugs and logistics from state, disbursement of drugs and logistics to drug administrators.

**Fig 1:**
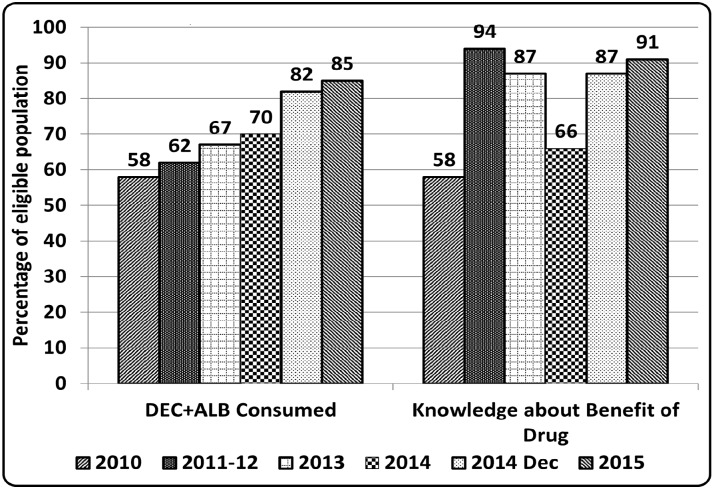
Directly Observed Treatment during MDA round (DOT-MDA) across years 2010–15. Years 2010, 2011–12, 2013, 2014, 2014 Dec, 2015.

Each year during the MDA round, 100 exit interviews of beneficiaries were conducted to directly observe the on-spot consumption of drug in all five districts. An increase from 58% to 85% in directly observed consumption and 58% to 94% in knowledge about benefits of the drug provided was seen across the years 2010 to 2015. (Fig 1) Post MDA survey was done to find program outcome indicators (coverage and compliance). Each year approximately 3000 people were studied leading to a total of 17551 people in six years, of which, approximately 96% were eligible for receiving drug according to the WHO criteria. The coverage rate was approximately 90% or more and drug (effective) coverage was more than 80% in all years. The epidemiological coverage (proportion of the at-risk population who consumed drug) was above 75% throughout reaching to a maximum level of 89% in year 2015. There was a coverage compliance gap (CCG) of 4% to 12% in various years. Further details are shown in Table 2.

**Table 2:** Coverage rate and compliance rate over last five years.

	Total Population	Eligible Population	Population Received Drug	Coverage Rate (%)	Population Consumed Drug	Compliance Rate (%)	Coverage Compliance Gap (CCG) (%)	Drug (Effective) Coverage (%)	Epidemiological Coverage (%)
2010	2815	2714	2421	89.2	2229	92.06	7.94	82.1	79.2
2011–12	3033	2877	2690	93.5	2342	87.06	12.94	81.4	77.2
2013	2984	2828	2672	94.5	2362	88.3	11.7	83.5	89.6
2014	2807	2681	2592	96.7	2355	90.9	9.1	87.7	83
2014DEC	2905	2808	2530	90.1	2354	93.1	6.9	83.3	81
2015	3007	2890	2681	92.7	2556	95.3	4.7	88.4	89.3

Approximately 10% or less population was left uncovered by MDA each year. The reasons for non-coverage were many and varied but mostly related to drug delivery system. (Table 3) The people did not get drug because either DA did not visit houses (3% to 53%) or they missed people (26.6% to 50.3%). In approximately 15% cases (range of 0–31.6%) DA did not give the drug to the eligible population by misclassifying them as non-eligible. Few people refused to accept drug (0–15%).

**Table 3:** Percent wise reasons for non-coverage.

Reason for Non-Coverage	2010	2011–12	2013	2014-Jan	2014 Dec	2015
	%	%	%	%	%	%
Team did not visit	52.9	3.6	8.3	34	23	24.4
Absent at the time of visit/Missed	26.6	34.9	27.1	50	43	50.3
DD did not give drug	2.8	25.8	31.6	0	24.9	3.3
Do not know	13.9	35.7	31.1	0	5.3	6.7
Refused to accept	3.8	0	1.9	16	3.8	15.3
**Total**	100.0	100.0	100.0	100.0	100.0	100.0

The compliance rate was more than 85% in all years but still a small proportion of the population in the range of 4% to 11% did not consume the drug (CCG). The reasons for non-compliance were usually people (consumer) related. (Table 4) People forgot to swallow the drug in the range of 6.8% to 62.5% while there was no specific reason in 1.6% to 33% cases. In 21.6% and 48.6% cases, people refused to swallow drug considering themselves healthy. We did not come across any resistance against the program from the community but on the other hand, people did not feel any perceived threat from filariasis.

**Table 4:** Percent wise reasons of non-compliance among those who received drug.

Reasons for non-compliance	2010	2011–12	2013	2014-Jan	2014 Dec	2015
For no reason	33.0	1.6	14.8	2.3	31.8	4.8
Fear of side effects	1.1	4.4	6.5	0	5.1	1.6
DEC misplaced	0.0	0.0	3.9	0	2.3	0
Forgot	24.9	62.5	22.9	28.4	6.8	23.2
Absent	9.7	4.7	15.8	7.6	13.8	33.6
Refused	28.6	22.4	25.8	48.6	36.2	21.6
Minor sickness	2.7	4.4	10.3	13.1	4.0	15.2
Total	100.0	100.0	100.0	100.0	100.0	100.0

**Fig 2:**
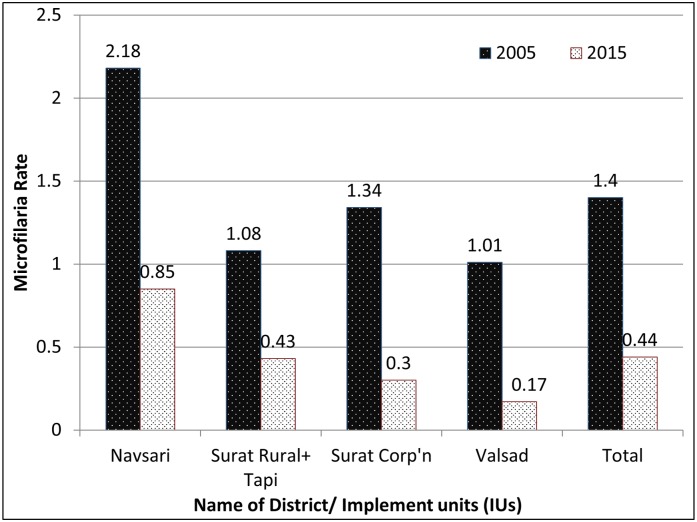
Baseline year 2005 and current year 2015 microfilariae rate. Year 2005 and 2015.

Approximately, 4000 nocturnal blood smears were collected before MDA round in each IU amounting to 20,000 blood slides per year to be examined for microfilaraemia to assess the impact of MDA. At the beginning of MDA in 2005 the Mf% was 1.4 which fell by 69% to 0.44 in 2015. There was a decrease of 60%, 61%, 70% and 83% in Surat and Tapi, Navsari, SMC and Valsad districts/ IUs respectively (Fig 2).

The microfilaria rate has decreased in all districts compared to baseline year 2005 as shown in Fig 2.

The Table 5 shows complete results of last six year pre- MDA night surveys in endemic districts (IUs) of Gujarat State. The MF rate gradually reached to less than one in all IUs in the year 2015. The current average Mf prevalence of South Gujarat region is 0.44. (Table 5)

**Table 5:** District-wise nocturnal blood smear results and Microfilaria (MF) rate.

Years	Slides Collected	Navsari	Surat Rural	SMC	Tapi	Valsad	Total
2010	Slides	4454	4085	4799	4178	4135	21651
	Positive	55	110	44	27	12	248
	MF Rate	1.23	2.69	0.92	0.65	0.29	1.15
2011	Slides	4218	4091	2106	3998	4074	18487
	Positive	28	159	0	18	30	235
	MF Rate	0.66	3.89	0	0.45	0.74	1.27
2012	Slides	4215	4069	6172	5121	4087	23664
	Positive	29	48	17	32	3	129
	MF Rate	0.69	1.18	0.26	0.62	0.07	0.55
2013	Slides	4225	4192	4150	4464	4109	21140
	Positive	27	82	0	48	37	194
	MF Rate	0.64	1.96	0	1.08	0.9	0.92
2014	Slides	4237	4199	4152	4040	4043	20671
	Positive	50	47	21	74	8	200
	MF Rate	1.18	1.12	0.51	1.83	0.2	0.97
2015	Slides	4118	4104	4042	3992	4067	20323
	Positive	35	25	12	10	7	89
	MF Rate	0.85	0.61	0.3	0.25	0.2	0.44
Total	Slides	25467	24740	25421	25793	24515	125936
	Positive	224	471	94	209	97	1095
Average	MF Rate	0.88	1.9	0.37	0.81	0.4	0.86

## Discussions

The GPELF is the largest public health intervention program attempted to date globally [3, 6, 11, 20]. MDA for ELF is one of the most challenging health programs considering the huge amount of treatments required to be administered in a small amount of time of three to four days [3, 6, 11, 20]. In South Gujarat region, approximately nine million (ninety lakhs in Indian terminology) treatments are administered every year. Though inbuilt supervision is an integral component of any program including MDA, monitoring of implementation by independent agencies is essential and warranted to strengthen and understand the success of strategies to combat filariasis.

The administration of simultaneous treatment to masses requires a huge amount of preparatory efforts in form of selection of drug administrators (DAs), training, micro-planning area, night surveys for microfilaraemia, advocacy and IEC for increasing awareness for compliance, drug procurement, delivery and management of any after affects [11, 12]. Pre MDA independent assessment of these activities at district /IU level ensured effective organization of drug delivery system especially the selection of DAs from local staff having a rapport with the community and their training to insist on-spot consumption. Pre MDA supervision made certain that supervisor health staff was nominated for every 5–10 DAs to support the MDA activity. Various studies on MDA coverage and compliance also underscore the importance of these activities particularly selection and training of DAs [7, 14, 20].

In the present study, during MDA round, treatments were directly observed (DOT) by team members. A total of six hundred DOTs were monitored in six years. A steady improvement in DOT was seen from 58% to 82% across the year 2010–2015. Increased awareness about benefits of the drug, up to 94%, was observed simultaneously. (Fig 1) We can safely assume that more knowledge about MDA leads to improved compliance which is in line with findings from other studies [7, 13, 20]. During MDA, it was seen that DAs and program managers were worried about side-effects of drug which made them reluctant for on-spot ingestion in people due to empty stomach. The reports from independent assessment consistently provided feedback and suggestions to increase compliance by insisting DOT by DAs. As the program progressed and fewer problems were encountered after consuming drug, DAs became confident to ask people to swallow drugs in front of them which may have led to increased compliance. (Fig 1) A systemic review done by Krentel of 79 studies across the world to find out factors influencing compliance for MDA confirms similar findings and suggest that directly observed MDA (DO- MDA) requires more efforts by drug delivery system [20]. Other studies that were done in India also confirm the role of DAs to insist on-spot ingestion to ensure compliance [4, 20].

During the post-MDA survey 17,551 people were interviewed in 120 clusters from the year 2010 to 2015. Comparing evidence from two systemic reviews on MDA done by Babu (36 papers) and Krentel (40 out of 79 articles), in Indian settings, the present study has covered maximum population and clusters [7, 20]. Approximately 96% population was eligible /target for MDA. Another study from Gujarat on MDA also reports 97.1% population as eligible [14]. Our survey reports high coverage (proportion of the eligible population who received the drug) and compliance rates above 85% in all years from 2010 to 2015. (Table 2) Both drug coverage (proportion of the eligible population who ingested the drug) and epidemiological coverage (proportion of the total population who ingested the drug) were above the recommended guidelines of 80% and 65% in all years [3, 6]. The coverage and compliance increased compared to previous years showing more reach to people by health care delivery system. A systematic review of 36 papers done on MDA in India by Babu, report coverage rates varied between 48.8% and 98.8%, while compliance rates ranged from 20.8% to 93.7%. They also report that the effective level of compliance ≥65%, was reported in only 10 of a total of 31 MDAs [7, 9, 14, 16, 22]. On the basis of the current survey, it can be concluded that overall coverage for ELF was adequate in South Gujarat region.

Though the present study reported good coverage and compliance rates from 2010 to 2015 after five initial rounds of MDA, there was some non-coverage and subsequent non-compliance which needs to be highlighted to implement strategies to improve the program and achieve elimination of LF by 2020. Approximately 5–10% people could not receive and 4% to 12% did not consume the drug. There was wide variation in reasons in different years (Tables 3 and 4) but still the most consistent were DAs related (did not visit, missed or absent people at home and did not give drug) for non-coverage and people’s attitude (no reason, absent, forgot or refused) for non-compliance. The indicators improved with awareness. These findings are in line with other research studies conducted in India and globally which report that drug provider and client attitude, previous experience with MDA and personal situation affects coverage and compliance [4, 7, 9, 14, 16, 20]. Our results also become important considering the fact that Babu et al in his systemic review reported that only 7 out of 36 studies on MDA studied reasons for non-coverage [7]. Emphasis on training, supervision with feedback and selection of DA from local health staff having a rapport with individuals for drug consumption can go a long way to improving compliance. Filariasis is a neglected tropical disease and there is no perceived threat to community. The attitude of people needs to be informed by national campaigns about filariasis and MDA.

Considering the huge task and costs involved in administering treatments, it is imperative to assess and decide end-points of MDA. The WHO guidelines require only monitoring of microfilaraemia to assess the impact of MDA. There are almost no longitudinal studies which compare coverage and impact indicator together [7, 13, 20]. The present communication studies and compares both coverage and impact indicator- Mf prevalence simultaneously. We found an overall 68% percent decrease in microfilaria rate from baseline year 2005 to 2015, similar to other studies [17, 23, 24]. (Fig 2 and Table 5) The evidence confirms that after 10 rounds of MDA, the LF transmission is interrupted and microfilaria rate has decreased to less than 1% in all IUs of Gujarat; threshold level recommended by WHO to provisionally stop MDA [17, 23, 24].

There were a few limitations to the present study. For determining the main outcome- coverage, the major part of study (other than DOT) depended on oral responses of people which are a major constraint for the studies related to compliance or people’s behavior. This was a long duration study and a large amount of data had to be collected every year from forty clusters within two weeks for coverage indicators and from forty sites for microfilaria rate, therefore, we needed multiple teams to collect data which could have introduced some recall and observer bias. Different studies have used different definitions for coverage indicators. To allow comparison, we tried to calculate all of the currently used indicators by WHO as well as by other studies and research community (Table 1) which could lead to some confusion for readers not familiar with different types of coverage [11, 13, 20]. There were wide variations in reasons for non-coverage and non-compliance which could have been due to different people’s and DAs attitude in different clusters or due to overlapping of various reasons of non-coverage and non-compliance by survey team members. The same sample size was studied for all IUs regardless of the difference in their characteristics, therefore, the results should be interpreted with caution.

The facts documented by this communication present important implication for program managers, policymakers, and drug delivery system involved in all health programs utilizing MDA or preventive chemotherapy strategy. The study outcomes and discussions suggest that implementing MDA program in large endemic areas gives good and sustained results comparable to small populations in form of decreased parasitaemia. Regular monitoring and supervision with continuous feedback by independent assessment help to target gaps in the effective implementation of the program.

Considering the huge task of providing mass treatments timely decisions are crucial to stopping MDA and step up surveillance to eliminate filariasis. This study provides evidence that South Gujarat region is ready for implementing surveillance in form of Transmission Assessment Survey (TAS) after discontinuation of MDA. The State fulfills all the criteria of implementing TAS by achieving above 65% coverage in ≥5 MDA rounds and demonstrating <1% microfilaraemia in each sentinel and spot-check site evaluated [25]. The TAS would help to, assess whether a series of MDA have successfully reduced the prevalence of infection to levels below predetermined threshold and provide the evidence base for program managers that MDA can be stopped.

Further research and verification in form of surveillance is essential to assure national government that national program have achieved their elimination goals [3, 17, 24].

This communication presents a detailed assessment of MDA across six years in a large geographical area. The program guidelines and indicators remained same for whole IUs as per the WHO guidelines. Therefore these results could be well generalized on at risk or endemic population for filariasis, which are currently implementing or have stopped MDA recently, with discussed limitations. The current study elaborates the path for elimination of lymphatic filariasis by documenting evidence to stop MDA and progress to TAS.

## Conclusions

Implementing MDA for elimination of LF gives sustainable results in large endemic populations if the epidemiological and drug coverage fulfills the required criteria. Results from independent assessment, performed regularly, could help in framing strategies to enhance the program. The impact indicator- microfilaria rate in all the districts and overall South Gujarat Region has reached and remained less than one percent signaling end-points of MDA. Post MDA stringent monitoring in form of TAS is recommended to keep vigil on maintenance of elimination received.

## Supporting information

S1 Excel Datasheet Post MDA survey data year 2010. (XLSX)

S2 Excel Datasheet Post MDA survey data year 2011–12. (XLSX)

S3 Excel Datasheet Post MDA survey data year 2013. (XLS)

S4 Excel Datasheet Post MDA survey data year 2014. (XLSX)

S5 Excel Datasheet Post MDA survey data year 2014 December. (XLSX)

S6 Excel Datasheet Post MDA survey data year 2015. (XLSX)

S7 Excel Datasheet Microfilaraemia survey results of Gujarat (2010–2015). (XLSX)
